# Identification of the Relationship between Hub Genes and Immune Cell Infiltration in Vascular Endothelial Cells of Proliferative Diabetic Retinopathy Using Bioinformatics Methods

**DOI:** 10.1155/2022/7231046

**Published:** 2022-02-03

**Authors:** Jing Huang, Qiong Zhou

**Affiliations:** Department of Ophthalmology, The First Affiliated Hospital of Nanchang University, Jiangxi Center of National Ocular Disease Clinical Research Center, Nanchang, 330006 Jiangxi, China

## Abstract

**Background:**

Diabetic retinopathy (DR) is a serious ophthalmopathy that causes blindness, especially in the proliferative stage. However, the pathogenesis of its effect on endothelial cells, especially its relationship with immune cell infiltration, remains unclear.

**Methods:**

The dataset GSE94019 was downloaded from the Gene Expression Omnibus (GEO) database to obtain DEGs. Through aggregate analyses such as Gene Ontology (GO) and Kyoto Encyclopedia of Gene and Genome (KEGG) pathway enrichment analysis, a protein-protein interaction (PPI) network was constructed to analyze the potential function of DEGs. Weighted gene coexpression network analysis (WGCNA) and Cytoscape software including molecular complex detection (MCODE) and cytoHubba plug-ins were used to comprehensively analyze and determine the hub genes. ImmuCellAI analysis was performed to further study the relationship between samples, hub genes, and 24 types of immune cell infiltration. Finally, gene-set enrichment analysis (GSEA) was employed to identify the enrichment of immune cell infiltration and endothelial cell phenotype modifications in GO biological processes (BP) based on the expression level of hub genes.

**Results:**

2393 DEGs were identified, of which 800 genes were downregulated, and 1593 genes were upregulated. The results of functional enrichment revealed that 1398 BP terms were significantly enriched in DEGs. Three hub genes, EEF1A1, RPL11, and RPS27A, which were identified by conjoint analysis using WGCNA and Cytoscape software, were positively correlated with the number of CD4 naive T cells and negatively correlated with the numbers of B cells. The number of CD4 naive T cells, T helper 2 (Th2) cells, and effector memory T (Tem) cells were significantly higher while CD8 naive T cells and B cells significantly were lower in the diabetic group than in the nondiabetic group.

**Conclusions:**

We unearthed the DEGs and Hub genes of endothelial cells related to the pathogenesis of PDR: EEF1A1, RPL11, and RPS27A, which are highly related to each other and participate in the specific biological process of inflammation-related immune cell infiltration and endothelial cell development, chemotaxis, and proliferation, thus providing new perspectives into the diagnosis of and potential “killing two birds with one stone” targeted therapy for PDR.

## 1. Introduction

According to the latest digital information from the International Diabetes Federation, there were an estimated 463 million diabetics among adults aged between 20 and 79 worldwide in 2019, and the number is expected to reach 642 million by 2040, with the pervasiveness rate increasing from 8.8% to 10.4%. In 2019, half (50.1%) of diabetics do not know they have the disease [1]. Diabetic retinopathy (DR) and DR-related blindness occur in 34.6% and 2.6%, respectively, among the total population of diabetic patients [2]. DR, especially proliferative diabetic retinopathy (PDR), makes a relatively minor contribution to vision deprivation or blindness in working-age people in developed countries [3], and its prevalence has been increasing in some developing countries with large populations [4, 5]. This has caused a heavy economic burden on all countries and has imposed serious obstacles to the development of global economic productivity [6].

DR is a neurovascular disease caused by the destruction of retinal neurovascular unit (NVU) due to diabetes, in which the lesions of intraocular microvessels are still dominant [7]. Chronic progressive diabetes leads to retinal microvascular leakage and occlusion and a series of secondary fundus lesions, such as microhemangioma, rigid exudation, cotton spots, neovascularization, vitreous proliferation, macular edema, and even retinal detachment [8]. The main risk factors for DR are the course of diabetes and blood glucose levels. Other important risk factors include HbA1c levels, blood pressure, serum total cholesterol, and low-density lipoprotein [8]. According to the pathogenesis of DR, nonproliferative diabetic retinopathy (NPDR) and PDR are the two major stages of DR observed in the clinic [9]. NPDR can be subdivided as mild, moderate, or severe based on fundus examination findings [9]. Approximately 16% of patients with moderate NPDR will progress to PDR within 5 years [10]. For very severe NPDR patients, the risk of developing PDR within one year is about 75% [11]. Once the disease progresses to PDR, the eye is 4.0 times more likely than an eye with mild NPDR to become blind within 2 years of DR diagnosis [12]. The main treatment methods are intravitreal injection of antiangiogenic drugs, macular and pan-retinal laser photocoagulation, and vitrectomy [13]. Antineovascularization therapies, mainly aimed at vascular endothelial growth factor (VEGF) and its receptors, have been the focus of DR treatment for many years. However, a considerable number of patients cannot benefit from these treatments [14], so there is an urgent need for new molecular targets and target-specific therapies.

Many studies have confirmed that hyperglycemia is the central precipitating factor in diabetic retinopathy [15–17]. Chronic injury and pathological changes to vascular endothelial cells are the core histopathological changes in DR injury [18, 19]. The effect of a hyperglycemic state on retinal vascular endothelial cells is multilayered and multifaceted. Retinal vascular endothelial cell function is affected by disrupting transmembrane glucose transport [20], extracellular matrix metabolism [21], protein transcription, and posttranscriptional regulation as well as cells' translation and posttranslational modification [22–24]. Abnormal function of vascular endothelial cells has been detected in the early stage of DR, and the damage is further aggravated by increased blood glucose [25]. In PDR, due to the breakdown of immune privilege and the destruction of the blood-retinal barrier (BRB), circulating immune cells will pass from retinal endothelial cells and infiltrate the retina and vitreous, causing retinal blood vessel damage which is characterized by chronic microvascular inflammation and neuronal damage [26–28]. Retinal vascular endothelial cells are the first vital station of circulating immune cell infiltration, and the overall immune infiltration situation is still unknown.

RNA sequencing (RNA-seq) is a technology that utilizes second-generation sequencing transcriptomics research methods to reveal a snapshot of the presence and quantity of RNA in a genome at a given moment [29]. It has been applied extensively as a powerful tool for exploring molecular mechanisms of diseases and identifying genome expression profiles [29]. The overall aim of this study was to use gene analysis technology to gain novel insights into the pathogenesis, diagnosis, prognosis, and treatment of DR. To achieve this aim, we adopted an RNA-seq dataset to determine differentially expressed genes (DEGs), filtrate potential biomarkers, analyze GO and KEGG pathways, and identify hub genes and their related immune cell infiltration abundances using the ImmuCellAI algorithm [30].

## 2. Materials and Methods

### 2.1. GEO Dataset Processing

The GEO database (https://www.ncbi.nlm.nih.gov/geo/) [31] was searched using “diabetic retinopathy, endothelial cell” as the subject term, yielding a total of 18 datasets. These data were filtered by selecting “expression profiling by high-throughput sequencing” as a study type and “Homo sapiens” as the organism, after which two datasets remained. After studying the specific details of the two datasets, dataset that did not meet the study criteria was discarded; we chose and downloaded GSE94019 which is based on the GPL11154 Illumina HiSeq 2000 (Homo sapiens) platform. The dataset GSE94019 contains a total of 13 human eye vascular endothelial cell samples, of which nine are from the fibrovascular membrane of patients with proliferative diabetic retinopathy, and four are from the retinas of people without diabetes. All samples were extracted, the data of each sample were preprocessed, and the average value at which the gene corresponded to multiple probes was recorded.

### 2.2. Analysis of Differential Expression

R software (version 4.1.0; https://www.r-project.org/) coupled with the Bioconductor package (http://www.bioconductor.org/) were implemented to correct and analyze the original data. RNA-seq data processing and normalization were performed using the edgeR package with trimmed-mean of *M* values (TMM) modus [32]. Criteria for statistical significance were logFC (fold change) > 2 and false discovery rate (FDR) < 0.05. DEGs, which are identified as genes that meet the screening criteria, were visualized on a heatmap by utilizing the pheatmap package and on a volcano plot by applying the EnhancedVolcano package.

### 2.3. Functional Enrichment Analysis

GO functional annotation and KEGG enrichment analysis were conducted on the DEGs, and results were visualized by implementing the R packages clusterProfiler, org.hs.eg.db, and enrichplot. The GO enrichment analysis consists mainly of biological processes (BPs), cellular components (CCs), and molecular functions (MFs). For the purposes of the present study, BP, which is the most important part of GO, was used for screening and analysis. The statistical significance criterion was set as adjust value of *p* < 0.05.

### 2.4. Coexpression Network Construction

The weighted gene coexpression network analysis (WGCNA) R package was used, and the gene expression profiles of all 17233 genes were occupied to reveal DR-related modules. Raw data were downloaded from the sample obtained from the GEO database. The edgeR package was used to process the data, and the upper quantile normalization modus was introduced for background amendment and standardization. Genes with standard deviation less than 0.5 times that of samples in the integrated dataset were eliminated, and other screened genes were introduced into WGCNA. To ensure that the network was scale-free, the pickSoftThreshold function was used to calculate the soft threshold of adjacency. The topological overlap matrix (TOM) was constructed after the adjacency matrix was transformed. By hierarchical clustering and use of a dynamic tree cutting function detection module, the genes with analogical expression portraits were allocated into several gene modules based on TOM dissimilitude measurement with the minimal unit of the gene tree dendrogram at 50, and the cutHeight value at 0.9. Module member (MM) and gene significance (GS) were calculated to reveal clinically related attributes. Each step of the above construction process is visualized by WGCNA and its related R packages. The genes and information contained in the module were retained for further analysis.

### 2.5. PPI Network Construction

A PPI network diagram of DEGs was developed using the STRING database (https://string-db.org/) with the maximum confidential interaction score of 0.99. Cytoscape software was used to optimize visualization of the network, and the MCODE plug-in was employed to analyze the PPI network including all DEGs to obtain the key clusters of the most closely interrelated genes. The network analyzer was used to calculate and analyze the score of each point in a multidimensional approach for the entire undirected network. In the MCODE plug-in setting, the original parameters (degree cutoff ≥ 2, node score cutoff ≥ 2, *K* − core ≥ 2, maximum depth = 100) were introduced to calculate and detect the key gene network model with intensive protein-protein relationship. All eleven algorithms including MCC, DMNC, MNC, Degree, EPC, EcCentricity, Closeness, Radiality, Betweenness, Stress, and BottleNeck in the CytoHubba plug-in were applied, and the intersection of the top 100 nodes from each method was recorded to identify the latent gene clusters. The gene cluster in the key module of WGCNA was intersected with the gene clusters obtained by MCODE and CytoHubba plug-ins in Cytoscape to obtain the final hub genes.

### 2.6. Immune Cell Infiltration Analysis

Immune Cell Abundance Identifier (ImmuCellAI) was used to estimate abundance of 24 immune cell types from RNA-seq digital gene expression data and obtain the immune cell infiltration matrix [30]. The landscapes of immune cell infiltration in retinal endothelial cells (RECs) from nondiabetic retinal tissue and fibrovascular membrane endothelial cells (FVMs) from PDR tissue were downloaded. The proportion of each immune cell subtype was extracted from the sample. A heatmap including the 24 immune cell types was created using the pheatmap package. A heatmap showing correlations between immune cells was plotted using the R package PerformanceAnalytics. The beanplot package was used to compare the distributions of the 24 immune cells between the REC and FVM groups. Lollipop plots showing the correlations between hub genes and immune cells, were created using the ggplot2 package, and the correlations were measured using the Pearson method.

### 2.7. GSEA of Hub Genes

The GSEA program was used to analyze genome-wide expression profiles from microarray data. Within this program, two reference database index files (c2.cp.kegg.v7.3.symbols.gmt and c5.go.bp.v7.3.symbols.gmt) were used to generate GSEA image sets. The multi-GSEA images in the BP of GO were redrawn using the clusterProfiler package in R [33]. Criteria for statistical significance were *p* < 0.05 and FDR *q* < 0.25.

### 2.8. Verification of Hub Genes

The software package pROC in R was used to analyze and assess the diagnostic value of hub genes using receiver-operating characteristic (ROC) curves. Criteria for hub genes were area under the ROC curve (AUC) greater than 0.80 and Wilcoxon test *p* < 0.05. The details of the datasets used for analysis in this study were presented in Table 1.

### 2.9. Statistical Analysis

All statistical analyses were calculated using R software (4.1.0) and associated packages as explained above.

## 3. Results

### 3.1. Identification of DEGs

**Figure 1:**
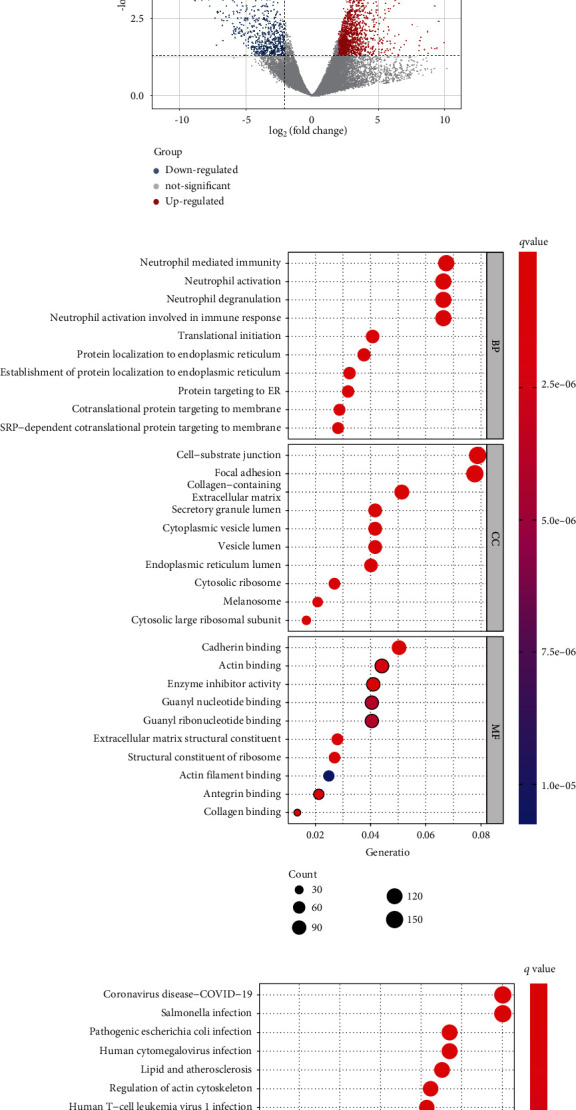
(a) DEG heatmap. The top 50 upregulated (red) and top 50 downregulated (blue) DEGs are shown, in order of FDR value from low to high. (b) DEG volcano plot showing the 20 most upregulated and downregulated genes. (c) GO bubble chart showing the top 10 terms manifested from the BP, CC, and MF directories. (d) KEGG bubble chart. Top 30 KEGG enrichment terms are shown.

Using the EdgeR package, a total of 2393 DEGs were identified, of which 1593 genes were upregulated and 800 were downregulated. DEGs were filtered and rendered at a set threshold and visualized by heatmap and volcano plot, in which heatmap illustrated the first 50 upregulated and downregulated genes (Figure 1(a)), while volcano map demonstrated the top 20 upregulated and downregulated genes (Figure 1(b)).

### 3.2. GO and KEGG Enrichment Analysis of DEGs

The functional enrichment results showed that 1398 BP terms, 178 CC terms and 130 MF terms were significantly enriched in DEGs. The BP of DEGs is mainly related to the activation and degranulation of neutrophil-related immune response, the initiation of protein translation, and the location of organelle assembly. Cell-substrate junction and focal adhesion are the two most abundant items in CC, while cadherin binding, actin binding, and enzyme inhibitory activity were the most abundant terms in MF (Figure 1(c)). In KEGG terms, in addition to some chronic and infectious disease pathways, diabetes mellitus and its complications and DR-related pathways such as tumor necrosis factor (TNF) signaling pathway, antigen processing and presentation, phototransduction, IL-17 signaling pathway, and AGE-RAGE signaling pathway in diabetic complications were significantly highly enriched. The results also illustrated significant enrichment in pathways in cellular processes such as phagosome, lysosome, regulation of actin cytoskeleton, focal adhesion, apoptosis, and pathways in genetic information processing such as ribosome, protein processing in endoplasmic reticulum (Figure 1(d)).

### 3.3. Gene Coexpression Web Development and Fundamental Module Recognition

**Figure 2:**
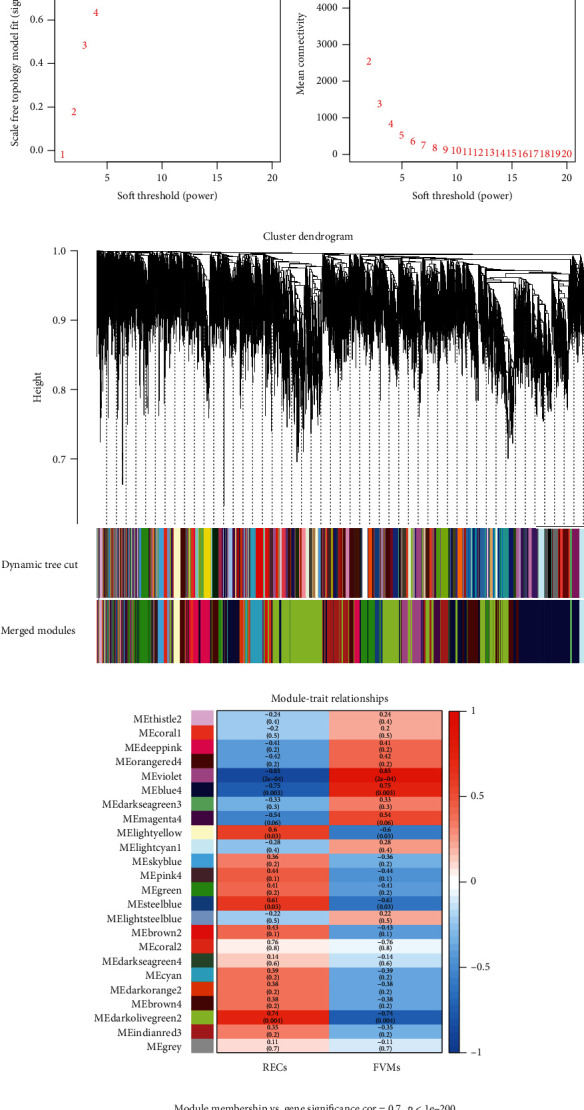
(a) Clustering of samples was executed to check outliers. All samples were subsumed in the cluster and were well classified, and no sample contains outliers. (b) Violin box plots show the expression profile distributions of all samples. (c) The soft threshold was calculated and determined using the pickSoftThreshold function. (d) A hierarchical clustering dendrogram was used to analyze coexpressed gene clusters, with each color representing a coexpression module. (e) Heatmap of the clinical distinguishing feature relevance of eigengenes, showing for each coexpression module gene cluster the correlation between the REC and FVM groups. (f, g) Scatter plots showing the correlations between MM and GS in the blue4 and violet modules.

When constructing the sample dendrogram, there were no outlier specimens, so no specimen was removed (Figure 2(a)). Through the analysis of sample gene expression distribution, it could be seen that the baseline distribution of sample gene expression was uniform and at the same level (Figure 2(b)). Using the pickSoftThreshold function, the optimal soft threshold was automatically set at 13, which makes the evaluation coefficient *R*^2^ of scale-free network run up to 0.9 for the first time (Figure 2(c)). After merging similar modules in the cluster tree with a cutting height of 0.3, 48 modules of genes with similar coexpression specificities were identified from the coexpression network (Figures 2(d) and 2(e)). The module eigengene (ME) in the violet module (*r* = −0.85; *p* = 2*e* − 4) displayed the highest negative correlation, while the ME in the blue4 module (*r* = −0.75; *p* = 0.003) showed the second highest negative correlation. These two modules were top two modules which were significantly correlated with clinical traits. Both the violet (cor = 0.75, *p* = 1.2*e* − 114) and blue4 (cor = 0.7, *p* < 1*e* − 200) modules showed significant positive correlations between MM and GS of the target gene (Figures 2(f) and 2(g))). Therefore, violet module and blue4 module were recognized as key modules.

### 3.4. PPI Network and Identification of Hub Genes

**Figure 3:**
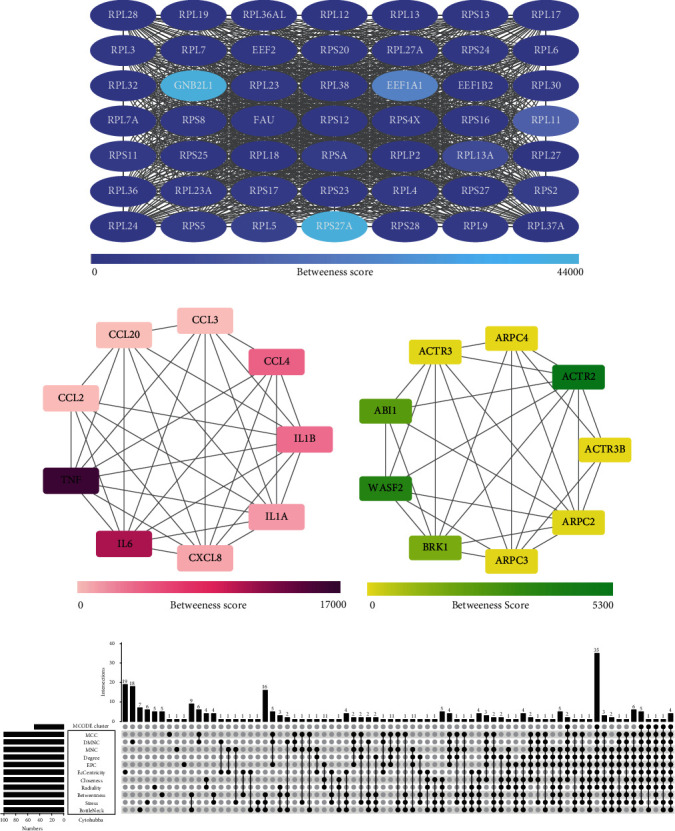
(a–c) Key gene modules predicted by MCODE. The top 3 key gene clusters calculated by the MCODE algorithm plug-in are uncovered, and their scores in the plug-in are 48.208, 8.250, and 7.250, respectively. The color in the figure from light to dark displayed how much the Betweenness Centrality score of each gene is. (d) The UpsetR plot unfurled the details of the number of overlapping genes obtained by the MCODE algorithm plug-in and the CytoHubba algorithm plug-in which 11 algorithms are employed.

After screening with a filter score of 0.99, the highest available in the string database, a network containing 789 nodes and 2755 edges was created using the cytoscape software. In the MCODE plug-in results, a total of 39 core networks were obtained, of which the three with the highest scores (48.208, 8.250, and 7.250) were extracted separately and visualized using Betweenness scores (Figures 3(a)–3(c)). The first of these with a score far exceeding other networks and the top 100 genes obtained by the eleven algorithms in CytoHubba were intersected using the upsetR method, and ultimately, a total of four candidate hub genes were obtained (Figure 3(d)). Intersection of these four genes with the gene sets of the two most critical modules obtained from WGCNA showed that two genes (EEF1A1, RPL11) were in the blue4 module and one gene (RPS27A) is in the violet module. After repeated screening, three genes, EEF1A1, RPL11, and RPS27A, were identified as hub genes.

### 3.5. Immune Cell Infiltration in RECs and FVMs

**Figure 4:**
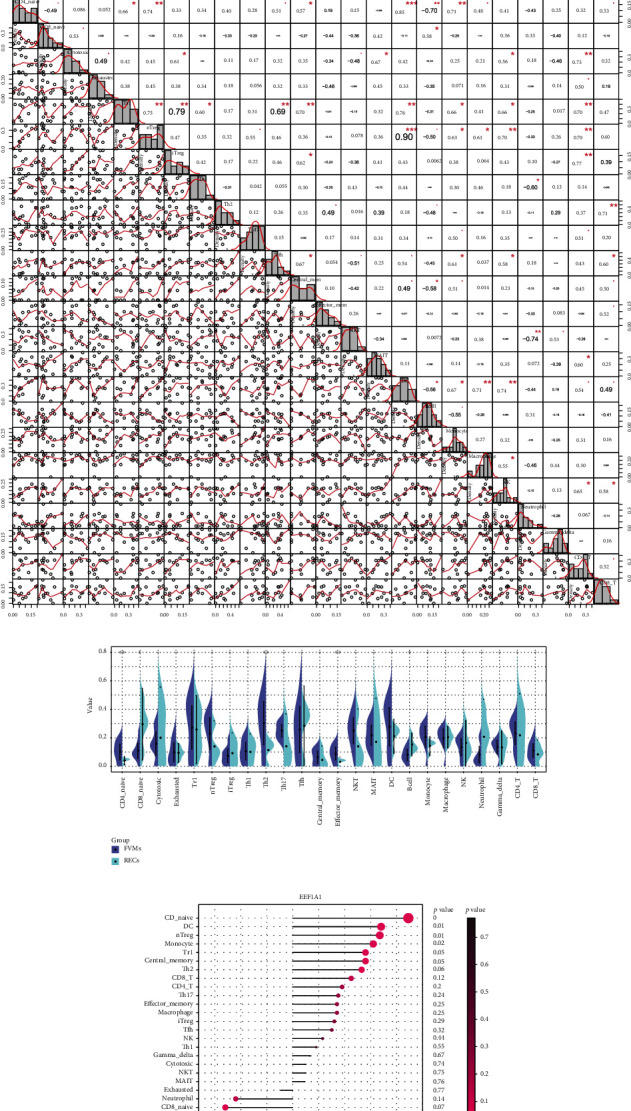
(a, b) Bar plot and heatmap of percentage distribution involved in 24 immune cell infiltrations in all samples. The upper part of the figure revealed the grouping of RECs and FVMs with square bars in different colors. (c) Correlation heatmap of 24 immune cells. The bottom left of the figure depicted the correlation distribution and linear regression between each immune cell subtype and all samples, and the top right described the correlation coefficient value and the value of *p* (^∗^ means *p* < 0.05, ^∗∗^ reveals *p* < 0.01, ^∗∗∗^ exhibits *p* < 0.001). (d) Bean plot. The distribution discrepancies of 24 kinds of immune cell subtypes between RECs and FVMs are exposed. ^∗∗^ in the figure indicated a significant increase in the FVMs group compared with the RECs group, and ^∗^ implied a significant increase in the RECs group compared with the FVMs group (*p* < 0.05). (e–g) Lollipop chart. Correlation between the hub genes EEF1A1, RPL11, and RPS27A and 24 kinds of immune cells are, respectively, unshrouded. The size of the dot represented the correlation coefficient between the hub gene and immune cells, and intensity of dot color represents the *p* value. (h) Box plot. Reflected the grouped expression of hub genes EEF1A1, RPL11, and RPS27A in the samples. The bar at the top of the diagram illustrated the *p* value of the difference between groups.

The ImmuCellAI algorithm was used to predict immune cell infiltration in the RECs and FVMs groups. Bar chart and heatmap show the relative percentage proportion of 24 immune cell types within the sample (Figures 4(a) and 4(b)). Absolute score differences between the two immune cell subgroups show that CD4 naïve cells, Th2 cells, and Tem cells in the FVMs group were significantly more advanced than in the RECs group, while the converse was true for CD8 naive cells and B cells, with significantly more cells in the RECs group than in FVMs group (Figure 4(d)). A significant correlation was found between DC and the other 11 types of immune cells; CD4 naïve cells, Tr1 cells, and CD4 T cells were significantly related to the other 10 types of immune cells; nTreg cells was significantly related to the other nine types of immune cells; Tfh cells, Tcm cells, Monocyte, NK cells, and CD8 T cells were significantly related to the other eight types of immune cells (Figure 4(c)). These correlations mean that one third or more of immune cell types were cross-correlated and may be considered as potential core immune cells with close links to the manifestation and improvement of diabetic retinopathy. Furthermore, three hub genes, the expression of which were considerably more advanced in the FVMs than the RECs group (EEF1A1, *p* = 0.0044; RPL11, *p* = 0.014; RPS27A, *p* = 0.0043) (Figure 4(h)), showed strong and significant positive correlation with CD4 naive, and a similar level of negative correlation with B cells (Figures 4(e)–4(g)). Thus, correlations exist between hub genes EEF1A1, RPL11, RPS27A, and immune cells, as well as between the different immune cell types.

### 3.6. GSEA Analysis Based on the Expression of Hub Genes

**Figure 5:**
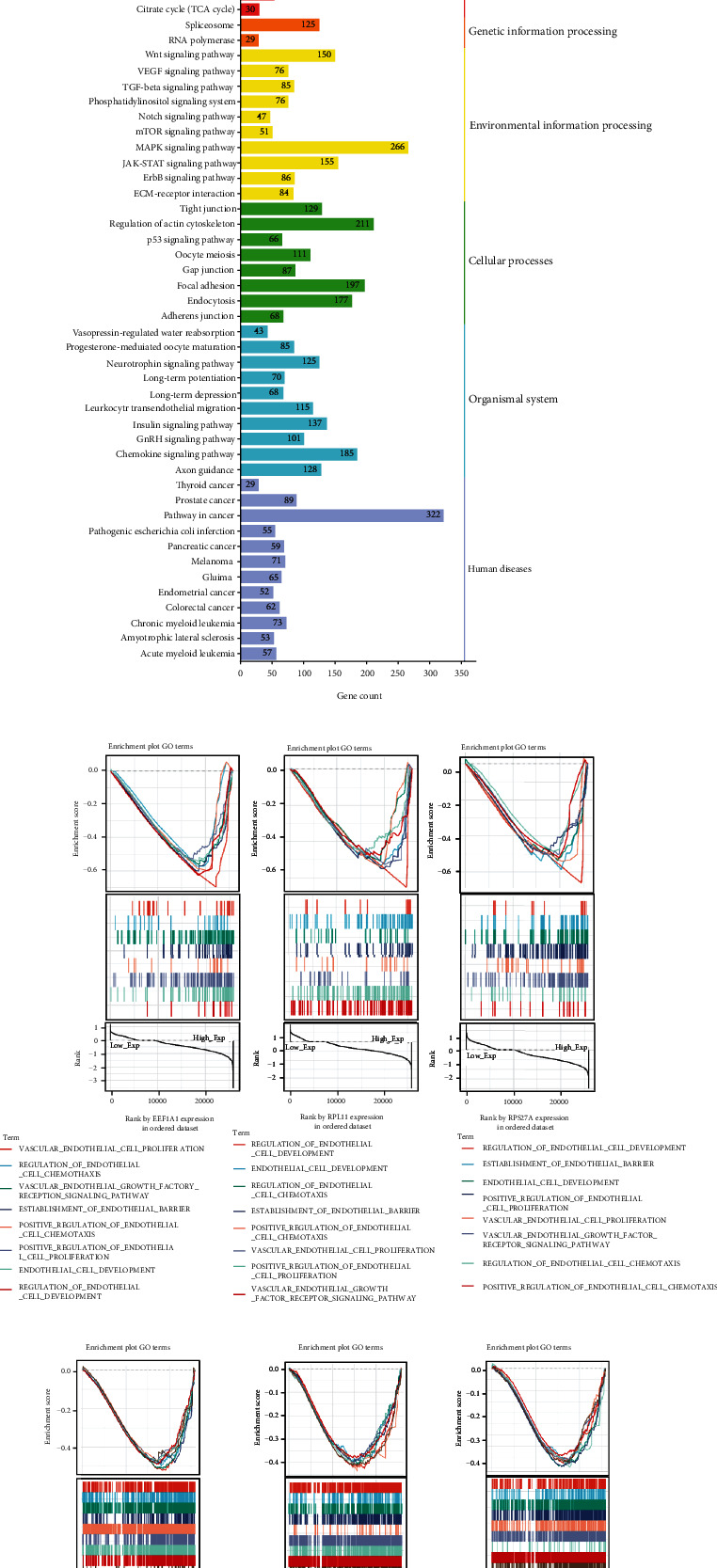
(a) The correlation heatmap of the hub genes EEF1A1, RPL11, and RPS27A. ^∗∗∗^ means *p* < 0.001. (b) Venn diagram. The intersection of KEGG terms of hub genes EEF1A1, RPL11, and RPS27A was manifested, including 47 common intersection items. (c) KEGG bar graph. The related terms are rearranged and classified according to the six classifications of KEGG pathways, and the length of the bar represented the number of gene count. (d, e) Multiple GSEA plot classified by the amount of hub gene expression. The former illustrated the enrichment of biological process pathways of GO related to endothelial cell phenotypic modifications, while the latter demonstrated the enrichment mode of endothelial cells participating in innate immunity and adaptive immunity.

Correlation analysis indicated that hub genes in this study have a strong relevance (Figure 5(a)), so when conducting GSEA analysis, the hub gene expression level was divided into two groups to observe any similarities and differences in the KEGG and GO analysis results under different gene expression levels, and the overlapping results are displayed (Figure 5(b)). GSEA analysis shows that for 47 of the 3 hub gene KEGG pathways were significantly more enriched in the high expression than low expression group (*p* < 0.05). Based on the KEGG pathway database, 47 important pathways were subdivided into six functional categories (Figure 5(c)). Some key pathways such as TGF-beta signaling, MAPK signaling, JAK-STAT signaling, VEGF signaling, citrate cycle (TCA cycle), and insulin signaling pathways have been indexed from the KEGG disease database to be closely related to diabetic retinopathy. Other pathways such as Wnt signaling, Notch signaling, mTOR signaling, ErbB signaling, ECM-receptor interaction, and GnRH signaling pathways require further research into interaction with the three hub genes. Research on the biological functions of GO analysis using GSEA has found that three hub genes are significantly and highly enriched in endothelial cell development and regulation, positive chemotaxis and regulation, proliferation and positive regulation, establishment of barrier, and VEGF receptor pathways in the high expression group (Figure 5(d)). Similarly, they are also highly enriched in the endothelial cell-related immune response pathways, such as the activation, differentiation, regulation of B cells and T cells, coupled with the activation as well as regulation of innate immune responses (Figure 5(e)).

### 3.7. Verification of Hub Genes Using GEO Database

**Figure 6:**
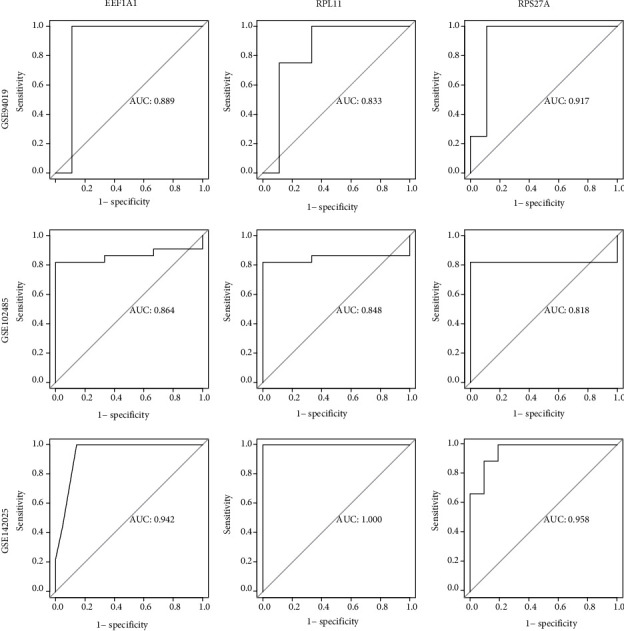
The ROC curve was applied to verify accurateness of hub genes. The corresponding hub gene name was presented at the top of the figure, and the GEO dataset number was manifested on the left side of the figure, where GSE941019 is the training set and GSE102485 and GSE142025 are the verification sets. The AUC of GSE941019 and GSE102485 was both greater than 0.8 and the AUC of GSE142025 was greater than 0.9.

The AUC of the training set GSE94019 and the validation set GSE102485 used in this study were both over 0.8, the AUC of the set GSE142025 exceeding 0.9 (Figure 6). These results show that the three hub genes have potential for high differentiation between RECs and FVMs and have good diagnostic value.

## 4. Discussion

While recent studies increasingly indicate that chronic low-grade inflammation and neurodegeneration of the retina are early manifestations of DR and play a role in its development, microangiopathy remains a dominant factor in this condition [34]. Endothelial dysfunction dominates the pathophysiology of microangiopathy in the diabetic retina and includes leukocyte adhesion, basement membrane thickening and pericyte loss, BRB damage, and neovascularization [35]. An increasing body of evidence illustrates that immune mechanisms are key to DR pathogenesis [34, 36]. Therefore, it is particularly important to fully understand the regulation of neovascularization and immune infiltration of vascular endothelial cells in DR.

Through annotation and functional enrichment analysis of DEGs, we found that DEGs are closely connected with immunoreaction and inflammatory signals, such as the innate immune response involving neutrophils, chemokine signal pathways, protein localization and transport and translation, cell-substrate adhesion, cell-mediated immunity, and apoptosis signaling pathways. During development of DR, increased retinal blood flow, abnormal leukocyte stasis, neutrophil as well as macrophage infiltration, alexine coupled with microglia activation, upregulation of cytokines, elevated vascular permeability and tissue edema, and the pathological manifestations of chronic retinal inflammation have been confirmed in animal models and DR patients [37, 38]. These objective findings are in accordance with the results of GO assay. Similarly, via KEGG analysis, we can clearly see that phagosomes, lysosomes, TNF signaling pathway, AGE-RAGE signaling pathway in diabetic complications, apoptosis, IL-17 signaling pathway, and others are also relevant to the chronic inflammatory pathological process of DR. Some studies have shown that photoreceptor cells are the source of diabetic retinal inflammatory proteins, and the release of soluble mediators can lead to the apoptosis of retinal endothelial cells [39], which is also consistent with the pathways obtained by KEGG.

After a series of complex model constructions and operation verifications, reliable hub genes EEF1A1, RPL11, and RPS27A were obtained. EEF1A1 gene, which encodes the same type of *α* subunit of complex named elongation factor-1, is responsible for aminoacyl tRNA enzymic transmission to ribosomes. EEF1A1 not only plays the leading role in protein translation prolongation, but also promotes cell growth and proliferation and inhibits apoptosis [40]. Studies have found that it also presides over protein posttranslational modification, protein degradation, and regulation of the cytoskeleton [41–43]. In the present study, the cytoscape plug-in MCODE showed that the gene cluster encoding ribosomal subunits in DEGs has the highest score, far exceeding that of other clusters, and contains 49 genes or their related genes. The RPL11 gene encodes 60s ribosome protein L11, which is an element of the 60s subunit and is related to the L5P ribosome proteins family. The RPS27A gene encodes 40s ribosome protein S27A, which is a constituent of the ribosomal 40s subunit and is a member of the S27AE ribosome protein family. Controlled alterations in ribosomal heterogeneity can upregulate or downregulate specific genetic networks [44]. When ribosomal proteins are reduced or eliminated, ribosomal biosynthesis is reduced or blocked, and in turn, the biosynthesis process is accelerated or increased [45]. The three hub genes identified in the present study are involved in the process of cellular translation including initiation, extension, termination, and posttranslation. They differ significantly between the two groups compared here, so they merit special attention and further investigation in the pathophysiological process of PDR.

Considering the importance of chronic inflammation and leukocyte stasis in the pathogenesis of PDR, ImmuCellAI was used to look for differences in distribution of immune cells between RECs and FVMs groups. The result is partly consistent with the results observed by Obasanmi et al. [46] and El Asrar et al. [47]. The immune cells with significant differences between the two groups are also core immune cells, which correlate strongly with most other immune cells. At or prior to the NPDR stage, one of the early events of diabetic retinal inflammation is leukocyte adhesion to the microvascular system. Increased leukocyte adhesion leads to the loss of endothelial cells and the destruction of the BRB [48, 49]. In the present study, neutrophils associated with vascular endothelial cells were lower in the FVMs group than in the RECs group, while other studies have shown that the level of neutrophils from blood in circumambient circulation was significantly higher in PDR than in a nondiabetic group [46, 50]. It has been demonstrated that the diseased vasculature releases a secretome that attracts neutrophils and triggers the production of neutrophil extracellular traps (NETs), which can clear diseased endothelial cells and reshape unhealthy blood vessels [51, 52]. In the PDR stage, especially in the inactive phase, due to the removal of neutrophils in the earlier stage, the abnormal intraocular endothelial cells were terminated, and the vascular remodeling was completed. The secretome of the abnormal vascular system was decreased, leading to a decrease in NETs, leading in turn to reduced attraction of neutrophils. At this point, the absolute number of vascular endothelial cells decreased, so the degree of neutrophil infiltration around the residual vascular endothelial cells decreased compared with the nondiabetic group. The significant downward regulation of B cells in the FVMs group compared with the RECs group may be due to the fact that most of the samples were in the inactive phase of PDR [53]. However, changes to several T cell subtypes require further investigation due to a lack of previous research data. We also found that correlations between the highly expressed genes EEF1A1, RPL11, RPS27A, and 24 immune cell types as well as the numbers of immune cell subtypes agreed with the differences in immune cell infiltration between the two groups. The above findings indicate that the three hub genes may be involved in the chronic inflammation and immune processes of PDR development.

Pathological retinal neovascularization and proliferation of endothelial cells are important features of PDR. The disorder of retinal metabolism and immune regulation in patients with diabetes induces glial cells to secrete a variety of inflammatory cytokines. Activated inflammatory mediators invoke more chemokines to act directly on endothelial cells through cascade amplification, which can not only participate in the expression of leukocyte recruitment but also be used as angiogenesis inducers of cytokines, chemokines, proinflammatory factors, and other factors [54]. Endothelial cells are affected by cytokines [55]. Upregulated proinflammatory cytokines may directly induce angiogenesis by binding to endothelial cells or indirectly induce angiogenesis by prompting endothelial cells to generate proangiogenic transmitter substances [56, 57]. In DR, retinal inflammation and vascular injury promote and regulate each other, resulting in the destruction of vascular structure and dissolution of the basement membrane, which eventually leads to destruction of the blood-retinal barrier. Endothelial injury induced by high glucose is mainly caused by cytokines released by endothelial cells, especially IL-1 *β*, TNF-*α*, and IFN-*γ* [58]. These cytokines can induce the expression of cytokines IL-8, MCP-1, and VEGF originated from endothelial cells [59]. Retinal ischemia and hypoxia has a strong stimulatory effect on endothelial cells [60]. Hypoxia is the most characteristic factor to promote expression of the VEGF gene [61]. When VEGF-A binds to VEGF-R2, it can promote the proliferation and exudation of vascular endothelial cells [62]. After VEGF-R2 is activated, the processes of tyrosine kinase receptor increases, leading to the activation of a variety of downstream signaling pathways and damage to the internal and external retinal barriers, important pathological mechanisms in the pathogenesis of diabetic macular edema (DME) [63]. In the present study, GSEA grouped by hub gene expression showed that the high-expression groups of three hub genes were highly enriched in biological processes such as the VEGF-R signaling pathway, endothelial cell development, chemotaxis, proliferation, and their respective regulation (chemotaxis and proliferation showed positive regulation). These findings are consistent with the pathological process and phenotypic transformations of endothelial cells in PDR, suggesting that high expression of the three hub genes was closely correlated with endothelial cell dysfunction and pathological alterations.

Our research has some limitations. First, although the study implemented WGCNA combined with Cytoscape software to conduct gene expression profiling on the RNA-seq dataset, the sample size was limited. Second, due to the lack of sufficient clinical information, including disease phenotypes and disease activity status, it was not possible to estimate the relationship between immune cells and disease severity. Thirdly, no in vivo experiments were conducted to verify these results. In future research, a large sample size combined with detailed clinical information is required for classification, and detailed studies of in vivo and in vitro pathogenesis at the molecular biology level will be required to verify our findings.

## 5. Conclusion

Using comprehensive bioinformatics analysis, we determined the differences in biological function of retinal vascular endothelial cells between PDR patients and normal samples. We found correlations between hub genes, EEF1A1, RPL11, and RPS27A and the immune response and infiltration of chronic inflammation of endothelial cells in PDR, as well as pathophysiological processes such as development, chemotaxis, and proliferation. These outcomes expand knowledge of the molecular mechanisms of endothelial cells in PDR and demonstrate their potential as new therapeutic targets for PDR.

## Figures and Tables

**Table 1:** Datasets implemented for analysis.

Dataset	Gene number	Platform	Case samples	Control samples
GSE941019	17233	GPL11154	9	4
GSE102485	14108	GPL18573	22	3
GSE142025	17184	GPL20301	21	9

## Data Availability

The datasets analyzed during the current study are available in Gene Expression Omnibus (https://www.ncbi.nlm.nih.gov/geo/).
